# The Power of Pain

**Published:** 1890-04-26

**Authors:** 


### Words of Consolation.

### THE POWER OF PAIN.

Pain is not a subject on which anything new can be said. In its simplest and lowest aspect, suffering is a punishment, but we all know that correction is a necessity for the ripening of men's characters. Some vices are swiftly punished with remorse and disease and shame in a measure disproportionate, it may seem, to the offences. We forget, however, that God does not see as man sees, but teaches by chastisement the real guilt of what we often are too ready to call trivial things. Pain is not only punishment, but also has a cleansing and corrective power. It elevates, refines, and dignifies the minds of men, Those who have great sorrows have the greatest influence in every walk of life. Our Saviour, Christ, the "Man of Sorrows," makes more impression on our hearts by His sufferings in soul and body than by the joys of His Resurrection, though without the latter His death pangs would have been incomplete for our redemption.

Christianity has taught us the uses of sorrow, and made every wise man content to bear his pain for the sake of the benefit which it brings with it. The Author and Finisher of our faith, for the joy that was set before Him, endured the Cross, despising the shame; and our light affliction, which is but for a moment, worketh for us a far more exceeding weight of glory, while we look not at the things which are seen but at the things which are unseen. Christianity bids us not wait till the sorrow comes, but to take up our Cross from the first and prepare ourselves for whatever may be sent upon us.

The perfect sinlessness, joined to the perfect suffering borne by our Saviour on the Cross, has cleared away for ever the idea that pain is always a punishment for sin. True it is that the bodily sins of a man are visited on himself as well as on his children, and though the suffering, of itself, is powerless to convert the sinner, it can and does prevent both the gratification and contagion of sin. It is often followed by remorse, which again brings repentance, and this refines and purifies the soul.

But though sin brings pain, suffering does not necessarily involve sin. You ask, Why, then, should the innocent suffer? For the benefit of themselves and others, we reply. Delicate health, privation, bereavement, may all refine the character and train the spiritual eye to that purity of heart which shall see God. So far from being our enemy it is our safest ally in the battle of life, and we fail if we shrink from the stern alliance.

Why should the sinless suffer? The pleasures of each generation pass away and leave no mark, but their sufferings help to build up the world. By pains and perils new generations are brought into the world; we live upon the death of the animals around us, and the necessities, the comforts, the luxuries of our existence are provided by the labour and sorrow of countless fellow men. Our freedom, our laws, our religion, have been won at the cost of broken hearts and noble lives laid down. Will we not, then, accept this state of things, and resolve that we, too, in our turn will spend and be spent for others, and find in it the deep truth of the benediction, "It is more blessed to give than to receive?"

